# 2,6-Dichloro-3-nitro­pyridine

**DOI:** 10.1107/S1600536811023683

**Published:** 2011-06-25

**Authors:** Hoong-Kun Fun, Suhana Arshad, B. Chandrakantha, Arun M. Isloor, Prakash Shetty

**Affiliations:** a X-ray Crystallography Unit, School of Physics, Universiti Sains Malaysia, 11800 USM, Penang, Malaysia; b Department of Chemistry, Manipal Institute of Technology, Manipal 576 104, India; c Department of Chemistry, National Institute of Technology-Karnataka, Surathkal, Mangalore 575 025, India; d Department of Printing, Manipal Institute of Technology, Manipal 576 104, India

## Abstract

The asymmetric unit of the title compound, C_5_H_2_Cl_2_N_2_O_2_, consists of two crystallographically independent mol­ecules. The pyridine ring in each mol­ecule is essentially planar, with maximum deviations of 0.004 (4) and 0.007 (4) Å. Short Cl⋯O [3.09 (3) and 3.13 (4) Å] and Cl⋯Cl [3.38 (12) Å] contacts were observed. No significant inter­molecular inter­actions were observed in the crystal packing.

## Related literature

For the role of the nitro­pyridine nucleus in the development of medicinal agents and in the field of agrochemicals, see: Davis *et al.* (1996 ▶). For the properties and use of pyridine derivatives, see: Vacher *et al.* (1998 ▶); Olah *et al.* (1980 ▶); Bare *et al.* (1989 ▶). For standard bond lengths, see: Allen *et al.* (1987 ▶). For the melting point, see: Johnson *et al.* (1967 ▶). 
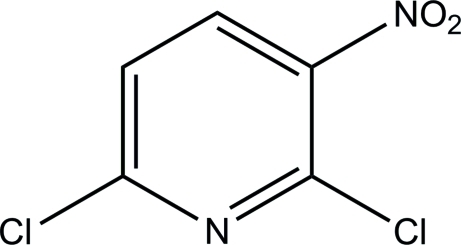


## Experimental

### 

#### Crystal data

C_5_H_2_Cl_2_N_2_O_2_
*M* *_r_* = 192.99
Monoclinic, 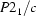

*a* = 7.9021 (8) Å
*b* = 19.166 (2) Å
*c* = 11.0987 (9) Å
β = 122.072 (5)°
*V* = 1424.4 (2) Å^3^
*Z* = 8
Mo *K*α radiation
μ = 0.85 mm^−1^
*T* = 296 K
0.40 × 0.27 × 0.24 mm

#### Data collection

Bruker SMART APEXII DUO CCD area-detector diffractometer
Absorption correction: multi-scan (*SADABS*; Bruker, 2009 ▶) *T*_min_ = 0.727, *T*_max_ = 0.821
16845 measured reflections
4817 independent reflections
2323 reflections with *I* > 2σ(*I*)
*R*_int_ = 0.060

#### Refinement

*R*[*F* ^2^ > 2σ(*F* ^2^)] = 0.067
*wR*(*F* ^2^) = 0.180
*S* = 1.08
4817 reflections
199 parameters
H-atom parameters constrained
Δρ_max_ = 0.55 e Å^−3^
Δρ_min_ = −0.42 e Å^−3^

### 

Data collection: *APEX2* (Bruker, 2009 ▶); cell refinement: *SAINT* (Bruker, 2009 ▶); data reduction: *SAINT*; program(s) used to solve structure: *SHELXTL* (Sheldrick, 2008 ▶); program(s) used to refine structure: *SHELXTL*; molecular graphics: *SHELXTL*; software used to prepare material for publication: *SHELXTL* and *PLATON* (Spek, 2009 ▶).

## Supplementary Material

Crystal structure: contains datablock(s) global, I. DOI: 10.1107/S1600536811023683/ng5183sup1.cif

Structure factors: contains datablock(s) I. DOI: 10.1107/S1600536811023683/ng5183Isup2.hkl

Supplementary material file. DOI: 10.1107/S1600536811023683/ng5183Isup3.cml

Additional supplementary materials:  crystallographic information; 3D view; checkCIF report

## Figures and Tables

**Fig. 1.**
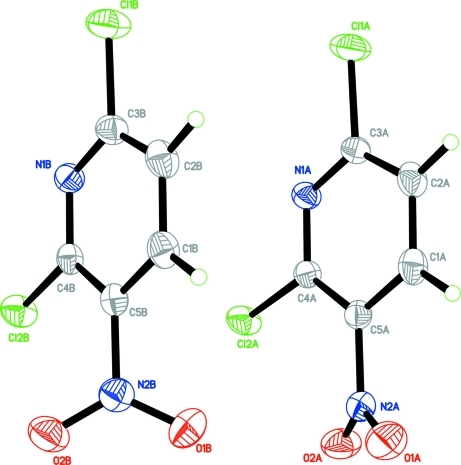
The molecular structure of the title compound, showing the two independent molecules with atom labels and 30% probability displacement ellipsoids.

**Fig. 2.**
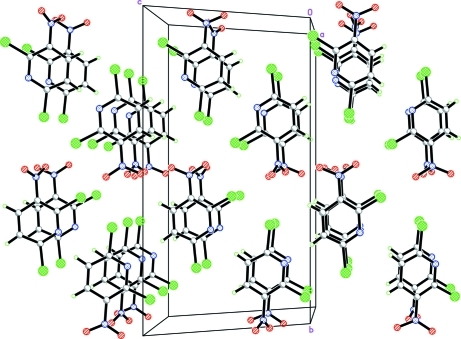
The crystal packing of the title compound.

## supplementary crystallographic information

### Comment

Nitropyridine nucleus played a pivotal role in the development of different medicinal agents and in the field of agrochemicals (Davis *et al.*, 1996). It is seen from the current literature that pyridine derivatives have been developed and used as insecticidal agents (Vacher *et al.*, 1998). Nitrated pyridines and their derivatives are important intermediates in synthesis of heterocyclic compounds in dyes and pharmaceutical products (Olah *et al.*, 1980). Fused heterocycles containing nitropyridine systems have been associated with several biological and medicinal activities including antiolytic (Olah *et al.*, 1980), antiviral and anti-inflammatory (Bare *et al.*, 1989) profiles.

The asymmetric unit of the tittle compound (Fig. 1), consists of two crystallographically independent molecules *A* and *B*. The pyridine rings (N1/C1–C5) for molecules *A* and *B* are essentially planar with maximum deviations of 0.004 (4) Å at atom C1A and 0.007 (4) Å at atom C3B, respectively. The bond lengths (Allen *et al.*, 1987) and angles are within normal ranges. In addition, short Cl···O [Cl1A···O2A (1 - *x*, -1/2 + *y*, 1/2 - *z*) = 3.093 (3) Å and Cl2A···O2A (1 - *x*, 2 - *y*, -*z*) = 3.132 (4) Å] and Cl···Cl [Cl2A···Cl2A (1 - *x*, 2 - *y*, -*z*) = 3.3839 (12) Å] contacts were observed.

The crystal packing is shown in Fig. 2. No significant intermolecular interactions were observed in the crystal packing.

### Experimental

2,6-Dichloropyridine (5 g, 0.033 mol) was added lotwise to mixture of concentrated H_2_SO_4_ (25 ml) and fuming nitric acid (10 ml) at 0 °C. After the addition, the reaction mixture was heated to 65 °C for 2 h. After completion of the reaction, the reaction mixture was cooled to room temperature and quenched with ice water. The solid that separated out was filtered and dried under vacuum. The crude product was purified by column chromatography using silica gel 60–120 mesh size and petroleum ether: ethyl acetate as eluent to afford title compound as a pale yellow solid. Yield: 3.0 g, 46.0%. *M.p.*: 333–338 K (Johnson *et al.*, 1967).

### Refinement

All H atoms were positioned geometrically [C–H = 0.93 Å] and refined using a riding model with *U*_iso_(H) = 1.2 *U*_eq_(C). There is no pseudo-symmetry in the crystal structure.

### Crystal data

**Table:**

C_5_H_2_Cl_2_N_2_O_2_	*F*(000) = 768
*M**_r_* = 192.99	*D*_x_ = 1.800 Mg m^−^^3^
Monoclinic, *P*2_1_/*c*	Mo *K*α radiation, λ = 0.71073 Å
Hall symbol: -P 2ybc	Cell parameters from 3419 reflections
*a* = 7.9021 (8) Å	θ = 2.4–31.7°
*b* = 19.166 (2) Å	µ = 0.85 mm^−^^1^
*c* = 11.0987 (9) Å	*T* = 296 K
β = 122.072 (5)°	Block, yellow
*V* = 1424.4 (2) Å^3^	0.40 × 0.27 × 0.24 mm
*Z* = 8	

### Data collection

**Table:**

Bruker SMART APEXII DUO CCD area-detector diffractometer	4817 independent reflections
Radiation source: fine-focus sealed tube	2323 reflections with *I* > 2σ(*I*)
graphite	*R*_int_ = 0.060
φ and ω scans	θ_max_ = 31.8°, θ_min_ = 2.4°
Absorption correction: multi-scan (*SADABS*; Bruker, 2009)	*h* = −11→11
*T*_min_ = 0.727, *T*_max_ = 0.821	*k* = −28→28
16845 measured reflections	*l* = −16→16

### Refinement

**Table:**

Refinement on *F*^2^	Primary atom site location: structure-invariant direct methods
Least-squares matrix: full	Secondary atom site location: difference Fourier map
*R*[*F*^2^ > 2σ(*F*^2^)] = 0.067	Hydrogen site location: inferred from neighbouring sites
*wR*(*F*^2^) = 0.180	H-atom parameters constrained
*S* = 1.08	*w* = 1/[σ^2^(*F*_o_^2^) + (0.0527*P*)^2^ + 1.2999*P*] where *P* = (*F*_o_^2^ + 2*F*_c_^2^)/3
4817 reflections	(Δ/σ)_max_ = 0.001
199 parameters	Δρ_max_ = 0.55 e Å^−^^3^
0 restraints	Δρ_min_ = −0.42 e Å^−^^3^

### Special details

**Table:**

Geometry. All e.s.d.'s (except the e.s.d. in the dihedral angle between two l.s. planes) are estimated using the full covariance matrix. The cell e.s.d.'s are taken into account individually in the estimation of e.s.d.'s in distances, angles and torsion angles; correlations between e.s.d.'s in cell parameters are only used when they are defined by crystal symmetry. An approximate (isotropic) treatment of cell e.s.d.'s is used for estimating e.s.d.'s involving l.s. planes.
Refinement. Refinement of *F*^2^ against ALL reflections. The weighted *R*-factor *wR* and goodness of fit *S* are based on *F*^2^, conventional *R*-factors *R* are based on *F*, with *F* set to zero for negative *F*^2^. The threshold expression of *F*^2^ > σ(*F*^2^) is used only for calculating *R*-factors(gt) *etc*. and is not relevant to the choice of reflections for refinement. *R*-factors based on *F*^2^ are statistically about twice as large as those based on *F*, and *R*- factors based on ALL data will be even larger.

### Fractional atomic coordinates and isotropic or equivalent isotropic displacement parameters (Å^2^)

**Table:**

	*x*	*y*	*z*	*U*_iso_*/*U*_eq_	
Cl1A	0.51344 (18)	0.68002 (5)	0.26085 (12)	0.0667 (3)	
Cl2A	0.49627 (16)	0.91466 (4)	0.03753 (9)	0.0540 (3)	
O1A	0.6135 (4)	1.00995 (14)	0.4256 (3)	0.0656 (8)	
O2A	0.4472 (5)	1.01996 (13)	0.1975 (3)	0.0641 (8)	
N1A	0.5042 (4)	0.80475 (13)	0.1709 (3)	0.0411 (6)	
N2A	0.5284 (4)	0.98557 (14)	0.3053 (3)	0.0433 (6)	
C1A	0.5404 (5)	0.87035 (18)	0.4058 (3)	0.0445 (8)	
H1A	0.5538	0.8926	0.4848	0.053*	
C2A	0.5356 (5)	0.79924 (18)	0.3979 (4)	0.0463 (8)	
H2A	0.5436	0.7720	0.4701	0.056*	
C3A	0.5182 (5)	0.76941 (15)	0.2786 (3)	0.0397 (7)	
C4A	0.5072 (5)	0.87406 (15)	0.1788 (3)	0.0359 (7)	
C5A	0.5251 (5)	0.90936 (15)	0.2945 (3)	0.0351 (7)	
Cl1B	1.01800 (19)	0.67408 (5)	0.25606 (12)	0.0673 (3)	
Cl2B	0.98563 (15)	0.91167 (5)	0.03234 (9)	0.0532 (3)	
O1B	0.9156 (5)	1.00415 (15)	0.3366 (3)	0.0731 (9)	
O2B	1.1216 (5)	1.01192 (14)	0.2658 (3)	0.0663 (8)	
N1B	1.0071 (4)	0.79906 (13)	0.1667 (3)	0.0403 (6)	
N2B	1.0196 (5)	0.97918 (15)	0.2975 (3)	0.0470 (7)	
C1B	1.0342 (5)	0.86410 (18)	0.4000 (3)	0.0450 (8)	
H1B	1.0422	0.8863	0.4773	0.054*	
C2B	1.0342 (5)	0.79259 (19)	0.3928 (4)	0.0481 (8)	
H2B	1.0421	0.7650	0.4646	0.058*	
C3B	1.0218 (5)	0.76313 (16)	0.2741 (3)	0.0427 (8)	
C4B	1.0086 (5)	0.86795 (16)	0.1754 (3)	0.0356 (7)	
C5B	1.0219 (5)	0.90270 (16)	0.2895 (3)	0.0376 (7)	

### Atomic displacement parameters (Å^2^)

**Table:**

	*U*^11^	*U*^22^	*U*^33^	*U*^12^	*U*^13^	*U*^23^
Cl1A	0.0972 (8)	0.0347 (4)	0.0768 (7)	0.0060 (5)	0.0521 (7)	0.0129 (4)
Cl2A	0.0868 (7)	0.0430 (4)	0.0416 (5)	−0.0036 (4)	0.0405 (5)	0.0053 (3)
O1A	0.085 (2)	0.0609 (17)	0.0551 (16)	−0.0130 (15)	0.0404 (16)	−0.0206 (13)
O2A	0.093 (2)	0.0406 (13)	0.0633 (17)	0.0096 (13)	0.0444 (17)	0.0094 (12)
N1A	0.0520 (18)	0.0343 (12)	0.0418 (15)	0.0003 (12)	0.0281 (14)	0.0016 (11)
N2A	0.0494 (17)	0.0377 (13)	0.0509 (17)	−0.0023 (12)	0.0321 (15)	−0.0046 (12)
C1A	0.049 (2)	0.056 (2)	0.0346 (17)	0.0028 (16)	0.0257 (17)	0.0035 (14)
C2A	0.050 (2)	0.055 (2)	0.0391 (18)	0.0067 (16)	0.0274 (17)	0.0137 (15)
C3A	0.0457 (19)	0.0331 (14)	0.0429 (18)	0.0047 (13)	0.0252 (17)	0.0086 (13)
C4A	0.0435 (19)	0.0332 (14)	0.0329 (16)	0.0010 (13)	0.0215 (15)	0.0025 (11)
C5A	0.0392 (18)	0.0337 (14)	0.0353 (16)	0.0015 (13)	0.0218 (14)	0.0012 (12)
Cl1B	0.0970 (9)	0.0390 (5)	0.0691 (7)	0.0050 (5)	0.0463 (6)	0.0105 (4)
Cl2B	0.0762 (7)	0.0501 (5)	0.0381 (4)	−0.0015 (4)	0.0335 (5)	0.0079 (3)
O1B	0.084 (2)	0.0648 (18)	0.093 (2)	0.0032 (15)	0.062 (2)	−0.0159 (16)
O2B	0.093 (2)	0.0517 (15)	0.0721 (18)	−0.0135 (15)	0.0556 (18)	−0.0038 (13)
N1B	0.0452 (16)	0.0392 (14)	0.0387 (15)	0.0028 (12)	0.0238 (13)	0.0050 (11)
N2B	0.0530 (18)	0.0447 (15)	0.0421 (16)	−0.0030 (14)	0.0244 (15)	−0.0050 (12)
C1B	0.051 (2)	0.0542 (19)	0.0355 (17)	−0.0033 (16)	0.0268 (17)	−0.0007 (14)
C2B	0.052 (2)	0.059 (2)	0.0369 (18)	−0.0006 (17)	0.0255 (17)	0.0104 (15)
C3B	0.046 (2)	0.0391 (16)	0.0417 (19)	0.0036 (14)	0.0224 (17)	0.0085 (14)
C4B	0.0360 (18)	0.0426 (16)	0.0296 (15)	0.0014 (13)	0.0183 (14)	0.0045 (12)
C5B	0.0385 (18)	0.0420 (16)	0.0358 (16)	−0.0011 (14)	0.0221 (15)	0.0013 (13)

### Geometric parameters (Å, °)

**Table:**

Cl1A—C3A	1.723 (3)	Cl1B—C3B	1.717 (3)
Cl2A—C4A	1.711 (3)	Cl2B—C4B	1.717 (3)
O1A—N2A	1.224 (3)	O1B—N2B	1.214 (4)
O2A—N2A	1.209 (4)	O2B—N2B	1.211 (4)
N1A—C4A	1.331 (4)	N1B—C4B	1.323 (4)
N1A—C3A	1.326 (4)	N1B—C3B	1.327 (4)
N2A—C5A	1.465 (4)	N2B—C5B	1.469 (4)
C1A—C2A	1.365 (5)	C1B—C2B	1.373 (5)
C1A—C5A	1.393 (4)	C1B—C5B	1.391 (4)
C1A—H1A	0.9300	C1B—H1B	0.9300
C2A—C3A	1.381 (5)	C2B—C3B	1.389 (5)
C2A—H2A	0.9300	C2B—H2B	0.9300
C4A—C5A	1.391 (4)	C4B—C5B	1.385 (4)
			
C4A—N1A—C3A	117.4 (3)	C4B—N1B—C3B	117.3 (3)
O2A—N2A—O1A	124.5 (3)	O2B—N2B—O1B	125.5 (3)
O2A—N2A—C5A	119.0 (3)	O2B—N2B—C5B	117.9 (3)
O1A—N2A—C5A	116.5 (3)	O1B—N2B—C5B	116.6 (3)
C2A—C1A—C5A	119.5 (3)	C2B—C1B—C5B	118.8 (3)
C2A—C1A—H1A	120.2	C2B—C1B—H1B	120.6
C5A—C1A—H1A	120.2	C5B—C1B—H1B	120.6
C1A—C2A—C3A	117.4 (3)	C1B—C2B—C3B	117.3 (3)
C1A—C2A—H2A	121.3	C1B—C2B—H2B	121.4
C3A—C2A—H2A	121.3	C3B—C2B—H2B	121.4
N1A—C3A—C2A	124.8 (3)	N1B—C3B—C2B	124.7 (3)
N1A—C3A—Cl1A	114.8 (2)	N1B—C3B—Cl1B	115.0 (3)
C2A—C3A—Cl1A	120.4 (2)	C2B—C3B—Cl1B	120.2 (3)
N1A—C4A—C5A	122.4 (3)	N1B—C4B—C5B	122.7 (3)
N1A—C4A—Cl2A	113.8 (2)	N1B—C4B—Cl2B	115.3 (2)
C5A—C4A—Cl2A	123.8 (2)	C5B—C4B—Cl2B	122.0 (2)
C1A—C5A—C4A	118.4 (3)	C4B—C5B—C1B	119.1 (3)
C1A—C5A—N2A	118.2 (3)	C4B—C5B—N2B	122.5 (3)
C4A—C5A—N2A	123.3 (3)	C1B—C5B—N2B	118.4 (3)
			
C5A—C1A—C2A—C3A	0.9 (5)	C5B—C1B—C2B—C3B	0.0 (5)
C4A—N1A—C3A—C2A	0.0 (5)	C4B—N1B—C3B—C2B	1.5 (5)
C4A—N1A—C3A—Cl1A	−180.0 (3)	C4B—N1B—C3B—Cl1B	179.7 (2)
C1A—C2A—C3A—N1A	−0.6 (6)	C1B—C2B—C3B—N1B	−1.1 (6)
C1A—C2A—C3A—Cl1A	179.4 (3)	C1B—C2B—C3B—Cl1B	−179.2 (3)
C3A—N1A—C4A—C5A	0.3 (5)	C3B—N1B—C4B—C5B	−0.9 (5)
C3A—N1A—C4A—Cl2A	178.0 (2)	C3B—N1B—C4B—Cl2B	−179.1 (2)
C2A—C1A—C5A—C4A	−0.6 (5)	N1B—C4B—C5B—C1B	0.0 (5)
C2A—C1A—C5A—N2A	179.3 (3)	Cl2B—C4B—C5B—C1B	178.1 (3)
N1A—C4A—C5A—C1A	0.0 (5)	N1B—C4B—C5B—N2B	−178.8 (3)
Cl2A—C4A—C5A—C1A	−177.5 (3)	Cl2B—C4B—C5B—N2B	−0.7 (5)
N1A—C4A—C5A—N2A	−179.9 (3)	C2B—C1B—C5B—C4B	0.5 (5)
Cl2A—C4A—C5A—N2A	2.6 (5)	C2B—C1B—C5B—N2B	179.3 (3)
O2A—N2A—C5A—C1A	−153.8 (3)	O2B—N2B—C5B—C4B	−44.8 (5)
O1A—N2A—C5A—C1A	25.6 (4)	O1B—N2B—C5B—C4B	135.7 (4)
O2A—N2A—C5A—C4A	26.1 (5)	O2B—N2B—C5B—C1B	136.4 (3)
O1A—N2A—C5A—C4A	−154.5 (3)	O1B—N2B—C5B—C1B	−43.0 (5)

## References

[bb1] Allen, F. H., Kennard, O., Watson, D. G., Brammer, L., Orpen, A. G. & Taylor, R. (1987). *J. Chem. Soc. Perkin Trans. 2*, pp. S1–19.

[bb2] Bare, T. M., McLaren, C. D., Campbell, D. J. B., Firor, J. W., Resch, J. F., Walters, C. P., Salama, A. I., Meiners, B. A. & Patel, J. B. (1989). *J. Med. Chem.* **32**, 2561–2573. 10.1021/jm00132a011 2573731

[bb4] Bruker (2009). *SADABS*, *APEX2* and *SAINT* Bruker AXS Inc., Madison, Wisconsin, USA.

[bb5] Davis, L., Olsen, G. E., Klein, J. T., Kapples, K. J., Huger, F. P., Smith, C. P., Petko, W. W., Cornfeldt, M. & Effland, R. C. (1996). *J. Med. Chem.* **39**, 582–587. 10.1021/jm950644v 8558530

[bb9] Johnson, C. D., Katritzky, A. R., Ridgewell, B. J. & Viney, M. (1967). *J. Chem. Soc. B.* pp. 1204–1210.

[bb6] Olah, G. A., Narang, S. C., Olah, J. A., Pearson, R. L. & Cupas, C. A. (1980). *J. Am. Chem. Soc.* **102**, 3507–3510.

[bb7] Sheldrick, G. M. (2008). *Acta Cryst.* A**64**, 112–122. 10.1107/S0108767307043930 18156677

[bb8] Spek, A. L. (2009). *Acta Cryst.* D**65**, 148–155. 10.1107/S090744490804362X PMC2631630 19171970

[bb3] Vacher, B., Bonnaud, B., Funes, P., Jubault, N., Koek, W., Assié, M.-B. & Cosi, C. (1998). *J. Med. Chem.* **41**, 5070–5083. 10.1021/jm9804329 9836623

